# GSK-3β, a double-edged sword in Nrf2 regulation: Implications for neurological dysfunction and disease

**DOI:** 10.12688/f1000research.15239.1

**Published:** 2018-07-10

**Authors:** Megan Culbreth, Michael Aschner

**Affiliations:** 1 Department of Environmental Medicine, University of Rochester School of Medicine and Dentistry, Rochester, NY, 14642, USA; 2 Department of Molecular Pharmacology, Albert Einstein College of Medicine, Bronx, NY, 10461, USA

**Keywords:** GSK-3β, Nrf2, oxidative stress

## Abstract

In the past decade, it has become evident that glycogen synthase kinase 3β (GSK-3β) modulates the nuclear factor erythroid 2-related factor 2 (Nrf2) oxidative stress response. GSK-3β functions as an inhibitor, both directly in the activation and indirectly in the post-induction of Nrf2. The incidence of oxidative stress in neurological dysfunction and disease has made this signaling pathway an attractive therapeutic target. There is minimal evidence, however, to support a distinctive function for GSK-3β mediated Nrf2 inhibition in nervous system decline, apart from the typical oxidative stress response. In both Alzheimer’s disease and brain ischemia, this pathway has been explored for potential benefits on disease etiology and advancement. Presently, it is unclear whether GSK-3β mediated Nrf2 inhibition markedly influences these disease states. Furthermore, the potential that each has unique function in neurodegenerative decline is unsubstantiated.

## Abbreviations

Alzheimer’s disease (AD), β-amyloid (Aβ), glycogen synthase kinase 3β (GSK-3β), middle cerebral artery occlusion and reperfusion (MCAO/R), nuclear factor erythroid 2-related factor 2 (Nrf2).

## Introduction

Glycogen synthase kinase 3β (GSK-3β) is a complex signaling molecule, involved in a variety of cellular processes ^1^. In the past decade, GSK-3β has been extensively investigated, particularly in regard to its role in neurological dysfunction and disease ^2, 3^. It is now evident GSK-3β participates in the cellular response to oxidative stress, a hallmark of several nervous system disorders. GSK-3β modulates this response through its interaction with nuclear factor erythroid 2-related factor 2 (Nrf2) ^4, 5^. Although the GSK-3β-Nrf2 association is well established, it has yet to be determined whether the dual regulatory nature of this interaction impacts disease advancement and potential therapies.

GSK-3β phosphorylates Nrf2, which results in its nuclear exclusion ^6^ and degradation ^4, 7, 8^. Interestingly, this was demonstrated to occur in a kelch-like ECH-associated protein 1 (Keap1)-independent manner ^4^; however, active GSK-3β was induced by hydrogen peroxide ^6^. Thus GSK-3β regulated Nrf2 activity possibly is oxidant-sensitive as well. GSK-3β has also been observed to indirectly modulate Nrf2 via Fyn phosphorylation. Fyn translocates to the nucleus resultant to GSK-3β phosphorylation, and in turn phosphorylates Nrf2, which stimulates its nuclear export ^5^. It must be noted that none of the above-mentioned reports were conducted in neuronal models. Moreover, observed effects were in transiently transfected cells, and not on endogenous protein.

Despite limited evidence that GSK-3β regulates Nrf2 in a similar manner in the nervous system, this signaling pathway has emerged as an attractive therapeutic target for neurodegenerative diseases. Oxidative stress is a well-established feature of several cognitive disorders, including Alzheimer’s disease (AD) and brain ischemia. However, a possible mechanism by which neurological dysfunction and disease modify GSK-3β mediated Nrf2 activity is unknown. Furthermore, the potential that this signaling axis contributes to disease etiology and progression has not been extensively explored.

Here, current research in AD and brain ischemia is highlighted with particular focus on potentially unique mechanisms by which these disorders modulate GSK-3β regulated Nrf2 activity.

## Alzheimer’s disease

GSK-3β is implicated in the advancement and potentially the etiology of AD. Extensive evidence corroborates its function in the learning and memory deficits observed in AD patients. Mechanistically, GSK-3β is linked to tau hyperphosphorylation and β-amyloid (Aβ) deposition, significant markers of AD pathogenesis. Furthermore, it modifies the oxidative stress response resultant to AD progression ^3^.

Recent reports have started to build connections between GSK-3β and Nrf2 in AD pathology and propose prospective therapies. GSK-3β suppression in a mouse model of AD was found to increase nuclear Nrf2 and total glutathione-S transferase (GST), an Nrf2 transcriptional target, in cortex. Moreover, reduced oxidative stress biomarkers and tau phosphorylation were observed ^9^. GSK-3β activation was also required for Aβ-induced axonal transport deficits detected in primary hippocampal neurons. Tau presence was necessary for this outcome, but intriguingly was Fyn-independent ^10^. Fyn phosphorylates tau ^11^ and Nrf2. The latter requires GSK-3β activation ^5^; however, GSK-3β directly phosphorylates Nrf2 as well ^6^. Irrespectively, Nrf2 is excluded from the nucleus as a result ^5, 6^. Potential associations between GSK-3β, Fyn, and Nrf2 were not demonstrated in this study ^10^. Furthermore, the Fyn-independent nature of Aβ and tau in AD could underscore distinct mechanisms by which GSK-3β activation mediates disease advancement.

A new AD therapy, which simultaneously inhibits GSK-3β and induces Nrf2 has been proposed. Interestingly, therapeutic Nrf2 activation was found to be GSK-3β inhibition-independent in the *in vitro* AD model. However, the potentially distinct beneficial properties of GSK-3β inhibition and Nrf2 induction were not examined. Tested compounds exhibited enhanced neuroprotection and reduced oxidative stress ^12^. Similar to the reports mentioned above, this study did not connect GSK-3β inhibited Nrf2 activation to GSK-3β activated tau hyperphosphorylation and Aβ deposition.

Current research on AD pathology has only just begun to elucidate a possible association between GSK-3β activation and the Nrf2-mediated oxidative stress response. Existing data does not, however, clearly demonstrate that GSK-3β activation represses Nrf2 function in AD models. Theoretically, observed oxidative stress in AD could occur entirely independent of GSK-3β. Moreover, other stress response factors apart from Nrf2 might be involved.

## Ischemia

GSK-3β has also emerged as a target in the treatment of brain ischemia. However, distinct from AD, recent studies have focused on GSK-3β regulation of Nrf2 in this disease state. Also unlike AD, an altered oxidative environment is the primary feature of brain ischemia, and not simply a secondary factor in the disease.

Existing evidence indicates GSK-3β inhibition enhances Nrf2 activity in the rat cerebral cortex following middle cerebral artery occlusion and reperfusion (MCAO/R). Interestingly, however, this brain ischemia model did not exhibit increased active GSK-3β and decreased nuclear Nrf2 simply as a consequence of MCAO/R. Furthermore, Nrf2 bound at the antioxidant response element (ARE) and subsequent target gene expression was only enhanced following MCAO/R in the presence of GSK-3β inhibitors ^13^. These data potentially imply GSK-3β regulation of Nrf2 is not directly involved in brain ischemia. Moreover, this study did not demonstrate improvement in the rat cerebral cortex following MCAO/R resultant to GSK-3β inhibition and Nrf2 activation.

Another report, however, did validate a GSK-3β inhibitor in an acute ischemia rat model, displaying reduced infarct volume, brain edema, and neurological deficit. These improvements were correlated with increased total Nrf2 protein and heme-oxygenase. Curiously, however, recovery was only examined 24 hours post-MCAO ^14^, whereas the article mentioned above showed GSK-3β inhibition effects as early as 6 hours post-MCAO ^13^. The timescale by which GSK-3β inhibitors exhibit therapeutic properties in brain ischemia must be further examined.

Present knowledge does not indicate brain ischemia directly alters GSK-3β regulation of Nrf2. Active GSK-3β is not enhanced in ischemia models, and Nrf2 downregulated as a consequence. Intriguingly, however, GSK-3β inhibitors do render improvement in these models, although evidence that Nrf2 is involved in this response is minimal. Possibly, GSK-3β inhibitors act on another signaling pathway in which GSK-3β is involved.

## Conclusion

The current body of evidence available to support a primary role for GSK-3β regulation of Nrf2 in neurological dysfunction and disease is incomplete. There is little to substantiate that this signaling axis is modulated differently under nervous system distress relative to a typical endogenous oxidative stress response. Moreover, the potentially distinct functions of GSK-3β and Nrf2 in cognitive disorders have not been linked to their possible combinatorial roles.

Polymorphisms in both the GSK-3β ^15^ and Nrf2 ^16^ gene promoter sequences have been associated with the onset of neurodegenerative diseases. There is no data, however, to validate that these polymorphisms impact the function of the GSK-3β and Nrf2 signaling pathway.

Future studies should focus on the distinctive functions for GSK-3β and Nrf2 in neurological dysfunction and disease. Based on these unique roles, potential links to their effects on each other should be formulated. Without these efforts, there is minimal evidence to substantiate a causative function for this signaling axis on cognitive disorder apart from an oxidative stress response. 

## Data availability

No data are associated with this article.
