# Supplementary material for: GSK-3β, a double-edged sword in Nrf2 regulation: Implications for neurological dysfunction and disease
Source: F1000Res. 2018 Jul 10;7:1043. [Version 1] doi: 10.12688/f1000research.15239.1 (PMC6053695; doi:10.12688/f1000research.15239.1)
Supplement: Peer review history

# Peer review: Referee response for version 1

**DOI:** 10.5256/f1000research.16599.r35941

**Published:** 2018-07-19

**Authors:** Alexey Tinkov

**Affiliations:** 1 Yaroslavl State University, Yaroslavl, Russian Federation; 2 RUDN University, Moscow, Russian Federation

The manuscript provides interesting data on the role of GSK-3b-Nrf2 interplay in neurological diseases. Although the authors review the existing data in relation to Alzheimer's disease and ischemic stroke, the mechanism may play a significant role in other disorders. It is also of particular interest whether this mechanism may be involved in mercury neurotoxicity, especially in view of the earlier study by the authors ( ^1^).

 Only minor changes may be recommended:

 It is recommended to add a reference to the sentence in the Introduction “Oxidative stress is a well-established feature of several cognitive disorders, including Alzheimer’s disease (AD) and brain ischemia”

 I suppose that the chemical nature of the compound family should be mentioned (2,4-dihydropyrano[2,3-c]pyrazoles)

I have read this submission. I believe that I have an appropriate level of expertise to confirm that it is of an acceptable scientific standard.

## References

[1] CulbrethMZhangZAschnerM: Methylmercury augments Nrf2 activity by downregulation of the Src family kinase Fyn. *Neurotoxicology*.2017;62: 10.1016/j.neuro.2017.07.028 200-206 10.1016/j.neuro.2017.07.028 28736149PMC5623621

# Peer review: Referee response for version 1

**DOI:** 10.5256/f1000research.16599.r35943

**Published:** 2018-07-13

**Authors:** Masatake Fujimura

**Affiliations:** 1 Department of Basic Medical Sciences, National Institute for Minamata Disease, Minamata, Japan

I am very interested in this report. This article was well written without any over discussion. Although the roles of GSK-3beta on neurological disease were well investigated for long time, that was not fully resolved at this time. Authors indicated the possibility that GSK-3beta modified the state of neurological disease through the regulation of Nfr2. In this regard, however, authors only mentioned  Alzheimer's disease and ischemia among neurological diseases. I hope that they discussed the roles of GSK-3beta through the regulation of Nfr2 in other neurological disease.

I have read this submission. I believe that I have an appropriate level of expertise to confirm that it is of an acceptable scientific standard.
